# Vacancy defect configurations in the metal–organic framework UiO-66: energetics and electronic structure†
†Electronic supplementary information (ESI) available: Further information on defect structures and band alignments, unit cell sizes and frontier orbital compositions. Optimised defect structures are available from DOI: 10.5281/zenodo.1064111. See DOI: 10.1039/c7ta11155j


**DOI:** 10.1039/c7ta11155j

**Published:** 2018-04-24

**Authors:** Katrine L. Svane, Jessica K. Bristow, Julian D. Gale, Aron Walsh

**Affiliations:** a Department of Chemistry , University of Bath , Bath , UK . Email: kasv@dtu.dk; b Curtin Institute for Computation , Department of Chemistry , Curtin University , PO Box U1987 , Perth , WA 6845 , Australia; c Department of Materials , Imperial College London , London , UK; d Department of Materials Science and Engineering , Yonsei University , Seoul 03722 , South Korea

## Abstract

Vacancy lattice sites in the metal–organic framework UiO-66 are known to have a profound effect on the material properties. Here we use density functional theory to compare the energies of defect arrangements containing missing linkers and missing metal clusters for different choices of charge compensation. Our results show that the preference for missing metal clusters or missing linker defects depends on the charge compensation as well as the overall concentration of defects in the crystal. Both regimes can be experimentally accessible depending on the synthesis conditions. We investigate the electronic structure of the different types of defects, showing that, despite some changes in the localisation of the frontier orbitals, the electronic energy levels are only weakly affected by the presence of point defects.

## Abstract 2

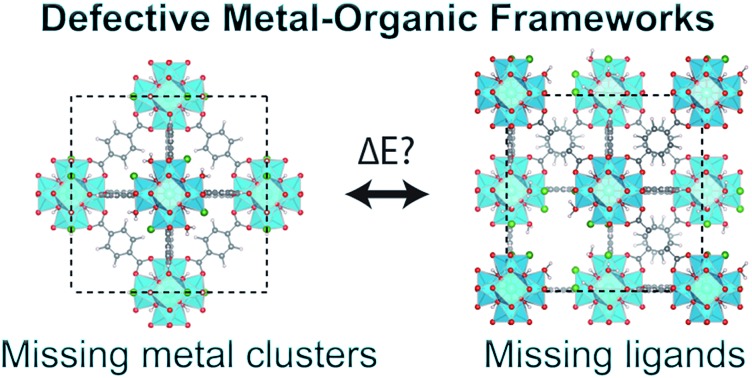
The energetics and electronic structure of defects in the metal–organic framework UiO-66 is investigated using density functional theory.

## 1 Introduction

Metal–organic frameworks (MOFs) are porous, crystalline materials made from metal clusters connected by organic linker molecules.^1^,^2^ The many possible combinations of metal and organic component leads to a wealth of diverse structures which have been investigated for potential applications ranging from gas-adsorption and catalysis,^3^,^4^ exploiting the porous nature of these materials, to sensing and photocatalysis which relies on their electronic properties.^5^,^6^

**Fig. 1:**
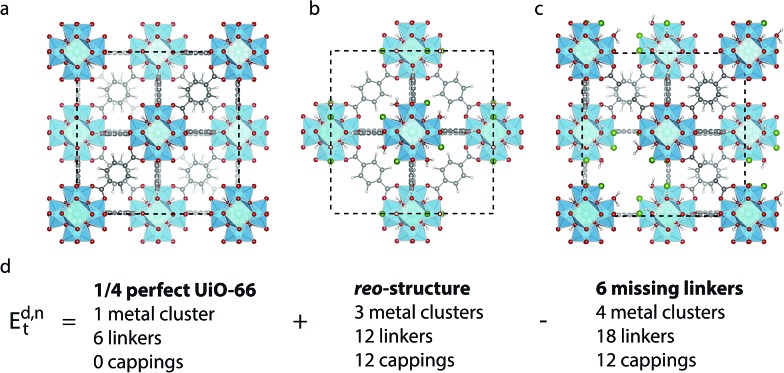
(a) Structure of defect-free UiO-66, (b) **reo**-structure containing missing metal cluster defects and (c) the 9_h_ structure with 6 missing linker defects. Zr is cyan, C is grey, O is red, H is white and Cl is dark green. (d) Scheme for comparing the energy of a structure with 6 missing linker defects and the **reo**-structure.

Recently, increasing attention has been focused on defects in MOFs, in recognition of their significant influence on the material properties as well as their surprisingly high concentration in some MOFs.^7^–^9^ A striking example is UiO-66 (ref. 10) (Fig. 1a), a popular MOF due to its chemical, mechanical and thermal stability.^11^–^14^ It consists of zirconium oxide clusters which, in the perfect crystalline structure, are connected to 12 benzene-1,4-dicarboxylate (BDC) linkers, however experiments suggest that between 1 and 4 of these linkers are missing from every cluster. The exact fraction of missing linkers varies depending on the synthesis method;^15^–^19^ in particular the addition of an acid modulator has been shown to increase the number of defects.^20^ This, in turn, leads to changes in the gas adsorption capacity,^18^,^21^–^23^ proton conductivity,^24^ catalytic activity,^25^–^29^ mechanical properties^19^,^30^–^32^ and chemical stability.^33^

Based on experiment, several different schemes for charge compensation of the metal cluster where a linker is missing have been suggested, including water and hydroxide,^12^,^34^–^36^ Cl^–^ (ref. 15 and 37) and small acids such as formate, benzoic acid and (trifluoro) acetic acid.^17^,^18^,^25^,^31^,^38^ These different charge compensation schemes are not necessarily inconsistent with each other, as the charge capping depends on the species available during synthesis. Furthermore, defects incorporated during synthesis can be removed by post-synthetic treatment. It is well known that the as-synthesised hydroxylated metal cluster (Zr_6_O_4_(OH)_4_)^12^,^35^ in a defect-free environment is dehydrogenated upon heating around 300 °C, resulting in two water molecules leaving to form Zr_6_O_6_.^12^,^29^,^37^ In a similar fashion the charge capping of a defect can be removed with one hydroxyl hydrogen,^25^,^29^,^37^ which in the case of four defects on the same cluster transforms the hydroxylated metal cluster into a dehydroxylated Zr_6_O_8_ cluster.^19^

Theoretical studies have investigated the structure and energetics of several of the defect configurations mentioned above.^26^,^29^,^32^,^39^,^40^ In all cases the formation of defects was found to be energetically unfavourable,^29^,^32^,^39^ with acid coordination having the lowest free energy of formation, followed by Cl^–^ (ref. 29) or Cl^–^/H_2_O.^39^ The probability of defect formation can be enhanced by increasing the capping : BDC ratio during synthesis.

The arrangement of defects on a larger scale has also been investigated. Reflections in an X-ray diffraction (XRD) pattern for UiO-66(Hf) synthesised with an acid modulator have been attributed to ordered structures of missing metal clusters with **reo**-topology (Fig. 1b), named the **reo**-structure in the following, which leaves the remaining metal clusters coordinated by only 8 linkers.^38^ A systematic study by Shearer *et al.* showed an increased intensity of the diffuse reflection with increasing amounts of acid modulator, and with decreasing p*K*_a_ values, which effectively corresponds to an increasing concentration of deprotonated modulator.^16^ The presence of diffuse reflections was further shown to correlate with a lower thermal stability of some samples,^12^,^16^ however it is also possible to synthesise a crystal where no such reflections are observed.^15^ This could suggest a lack of ordering of the missing clusters at low defect concentrations (*i.e.* small domain size), or simply that a different arrangement of defects is preferred in this situation. The energetics of different arrangements of missing linkers around dehydroxylated metal clusters has been investigated computationally,^26^,^32^ however to our knowledge no studies comparing the energetics of missing linker and missing metal cluster defects have been performed.

Here we use density functional theory (DFT) to investigate the energetics of different defect arrangements in UiO-66. We present a scheme for calculating the energetic preference for missing metal clusters relative to missing linkers only. Furthermore, we investigate the effect of the different types of defects on the band gap and frontier orbital localisations. Previous investigations have shown that the length of the linker in the UiO-family of materials and the addition of functional groups to the linker can change the electronic properties of the material,^41^–^43^ and it is therefore interesting to see if defects can have similar effects. We consider defects capped by trifluoroacetic acid (**tfaa**, CF_3_COO^–^), acetic acid (**aa**, CH_3_COO^–^), formic acid (**fa**, HCOO^–^), Cl^–^/H_2_O (**cl**) as well as defects compensated by dehydroxylation of the metal cluster (**e**). The first four cappings are chosen because they are likely to be present in solution during synthesis, and remain in the structure after drying under mild conditions. **tfaa**, **aa** and **fa** are commonly used as modulators to enhance the formation of defects during synthesis, while Cl^–^ is present when ZrCl_4_ is used as the Zr source and when HCl is added to the synthesis solution. The **e** defect only arises upon heating of the structure after synthesis, or as a short-lived state in solution.

## 2 Methods

### 2.1 Density functional theory calculations

The Vienna Ab initio Simulation Package (VASP) code^44^ was used to perform calculations under the Kohn–Sham density functional theory (DFT) framework. The PBEsol exchange–correlation functional^45^ with D3 dispersion correction^46^ was used for structure optimisation, using the projector augmented wave method for interaction between core and valence electrons of all atoms in the system. Forces were converged to 0.01 eV Å^–1^ and SCF convergence was set to 10^–8^ eV, with a plane wave basis set cutoff of 500 eV. Owing to the dimensions of the unit cell of UiO-66, *Γ*-point sampling of the Brillouin zone was found to be sufficient. This is also justified by the almost flat bandstructure of the material (*c.f.* ESI†).

For the parent UiO-66 structure the phonon spectrum was calculated to verify that the structure is a local minimum (*i.e.* has no imaginary frequencies), but calculation of phonons for the defective structures where symmetry is (partially) broken would be computationally unfeasible. We thus compare the enthalpies rather than the free energies in the following, however in previous investigations the influence of entropy on different arrangements with the same number of defects was found to be modest.^32^

The unit cell volume was optimised for all structures, since the presence of defects can lead to strain in the material. We note that comparison of energies across cells that are not the same size can lead to small errors, which should vanish in the limit of a large energy cutoff. We compared the energy difference between two different defect arrangements (*E*d,*n*t, see Section 3) for the **aa** capping calculated with a 500 eV and a 600 eV cutoff and found the difference to be less than 10 meV, thus this effect is expected to be small.

Single-point HSE06 (ref. 47 and 48) hybrid DFT calculations were carried out on the optimised geometries of UiO-66 and its defective structures for calculating electronic densities of states (DOS), optical band gap values and frontier orbital compositions. Band alignment was performed following the method of Butler *et al.*, using a plateau in the electrostatic potential in the centre of the MOF pores to align the vacuum potentials of each system.^49^,^50^ The average of the electrostatic potential (*Φ*_av_(*r*)) is calculated within a defined cube centered at *r* following the equation:1
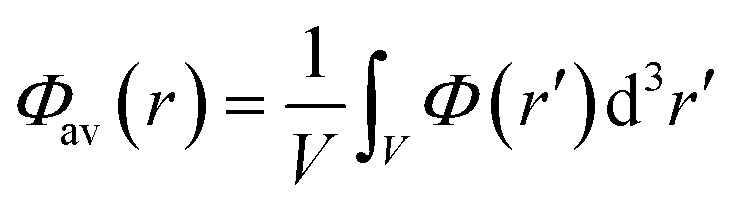



The variance of the electrostatic potential in the sampled volume in the centre of the MOF pores are given in the ESI,† demonstrating that the sampled points correspond to a plateau in the electrostatic potential of the systems. The side length of the cube used for calculating *Φ*_av_(*r*) was set to 0.75 Å.

## 3 Results

### 3.1 Defect energetics

All calculations are performed in the cubic unit cell which contains 4 metal clusters and 24 linkers for the defect-free structure (Fig. 1a), and the metal clusters are fully hydroxylated except when **e** defects are present, in which case a hydrogen is removed from the metal cluster for each missing linker.

We now want to investigate if a structure with only missing linkers is energetically favoured over the **reo**-structure. Starting with a given defect concentration corresponding to *n* missing linkers of type d in the cubic unit cell, it is possible to convert this structure into the **reo**-structure, which contains missing metal cluster defects, and an amount of defect-free UiO-66. The energy required for this transformation, *E*d,*n*t can be calculated following the equation:2
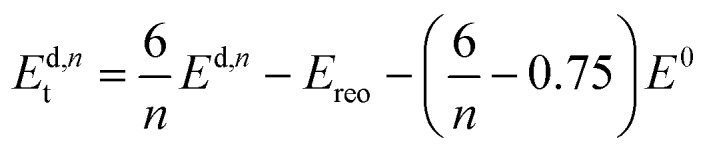
where *E*^d,*n*^ is the total energy of the structure with *n* missing linkers of type d, *E*_reo_ is the energy of the **reo**-structure and *E*^0^ is the energy of the defect-free structure. Eqn (2) ensures that the amount of metal, and the number of linkers and capping molecules balance, avoiding the need to calculate solvation energies of the species. An example of how this works for a defect concentration of 25% (*i.e.* 6 of the 24 linkers in the cubic unit cell missing) is shown in Fig. 1d.

We have calculated *E*d,1t and *E*d,6t for the five different types of cappings, corresponding to a low defect concentration of 4% and a more realistic concentration of 25%, respectively. The calculation with one defect is straightforward, since there is only one symmetry-inequivalent configuration of one defect in the cubic unit cell, however to get an accurate value of the transformation energy with 6 defects we must in principle first identify the lowest energy configuration of 6 missing linkers. Due to the many possible combinations in this case, it is beyond our computational resources to calculate all the possibilities. Instead we consider two different structures for each capping; one in which each metal cluster has three defects next to each other and the defects are clustered to form a missing tetrahedral cage, and one where the defects are dispersed and each metal cluster has three defects as far from each other as possible (*c.f.* ESI† for schematics of these structures). Since the two structures represent the limiting cases of clustered and dispersed defects, we would expect an energy difference if there are attractive or repulsive interactions between neighbouring defects. Following the naming convention in ref. 26 the structures would be named (9_h_,9_h_,9_h_,9_h_)_111111111111222_ and (9_c_,9_c_,9_c_,9_c_)_333333333333222_, respectively. We denote them by 9_h_ (missing tetrahedral cage) and 9_c_ (dispersed defects) in the following.

**Fig. 2:**
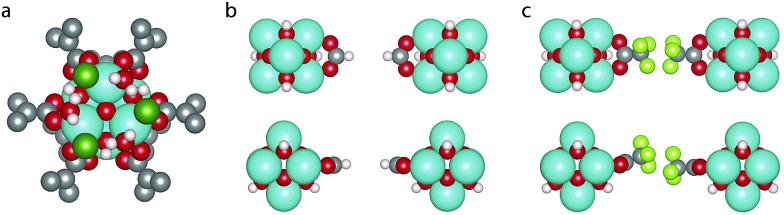
(a) Hydrogen bonding pattern between the Cl atoms and H_2_O molecules of a 9_h_ node with the **cl** capping and top and side views of (b) the **fa** capping and (c) the **tfaa** capping. Zr is coloured cyan, C is grey, O is red, H is white, Cl is dark green and F is light green.

The difference in energy between the two defect arrangements, Δ*E*_6def_ = *E*_9h_ – *E*_9c_, is given in Table 1, a negative value indicating a preference for the clustered 9_h_ arrangement. It can be seen that the **cl** capping shows a strong preference for the clustered defects, which arises because it allows the water molecules to make hydrogen bonds with two neighbouring Cl atoms (*c.f.*Fig. 2a). For the other cappings the numerical value of Δ*E*_6def_ is much smaller, suggesting that the relative positions of defects have a limited impact on the energy, as has also been found in previous studies comparing different defect arrangements.^26^,^32^,^39^ We have used the lower energy value for our calculations of *E*d,*n*t.

**Table 1:** Energy difference between the two investigated configurations with 6 missing linkers (Δ*E*_6def_), transformation energy for 1 and 6 missing linkers (*E*d,1t and *E*d,6t) and p*K*_a_ value for the acid modulators

Capping	**e**	**cl**	**fa**	**aa**	**tfaa**
Δ*E*_6def_ [eV]	0.17	–1.26	–0.03	0.10	0.73
*E* d,1 t [eV]	–0.90	2.17	1.89	1.81	–0.71
*E* d,6 t [eV]	–1.72	1.31	0.24	0.05	–0.82
p*K*_a_	—	—	3.77	4.76	0.23

The calculated values of the transformation energy are shown in Table 1, for the different choices of charge cappings. Negative values indicate that formation of the **reo**-structure is preferred, while positive values indicate that a structure with only missing linkers is preferred. The results show that the preferred defect structure depends on which capping agents are available from the synthesis solution, as well as the total number of defects. The **cl**, **fa** and **aa** cappings shows a clear preference for missing linkers at low defect concentrations. For **fa** and **aa***E*d,6t is small, indicating that both types of defects could be present at higher defect concentrations. For the **tfaa** and **e** defects formation of the **reo**-structure is preferred, however we note that **e** defects are formed from a structure with capped defects and the defect arrangement is thus expected to be inherited from this parent structure. In ref. 16 the total amount of missing linker defects obtained from thermogravimetric analysis (TGA) was compared to the intensity of the XRD-reflection that characterises the **reo**-structure. While the data is too sparse to be conclusive it seems plausible that there are differences in the relative amount of **reo**-structure for different types of modulator, and in particular **tfaa** appears to give rise to the largest relative amount of **reo**-structure.

To understand the different preferences for the different cappings we look at the optimised defect structures. Fig. 2b and c shows a missing linker defect with the **fa** and **tfaa** cappings. In the **fa** defect (Fig. 2b) the two cappings point directly towards each other and the unit cell dimensions in the plane of the defect are only slightly changed from 20.74 Å in defect-free UiO-66 to 20.76 Å (see ESI† for unit cell parameters of all optimised structures and comparison with experimental values). For **tfaa** we investigated two different structures of the single defect, one in which the **tfaa** cappings point directly towards each other, and one where they are misaligned as seen in Fig. 2c. In both cases the defect leads to an expansion of the unit cell in the plane of the defect to an average of 20.85/20.86 Å for the misaligned/aligned defect. This suggests a steric repulsion between the two cappings as can also be imagined by considering the proximity of atoms in Fig. 2c. Such steric repulsions are only present for a missing linker defect and not in the **reo**-structure where the distance between opposing cappings is larger, and indeed the **reo**-structure is found to have lattice parameters of 20.71 Å, close to the value for the defect-free UiO-66. We note that the misaligned **tfaa** single defect is only 36 meV more stable than the aligned defect, suggesting a soft energy landscape due to the balance between steric repulsion between the **tfaa** cappings and angular strain when bending the cappings away from the planar configuration. We therefore used several different starting structures including aligned and misaligned configurations for the **tfaa** structures containing missing ligand(s), however a complete investigation of all possible relative defect misalignments when multiple defects are involved was judged to be computationally unfeasible.

The **aa** capping is similar in size to the **tfaa** capping and we have treated it in a similar manner, considering both aligned and misaligned capping arrangements. Again, a small energetic preference for the misaligned configuration is found, however for this capping the missing linker and missing metal cluster structures are almost equal in energy, suggesting a smaller steric effect, as would be expected since the hydrogen atoms have a lower electron density than the fluorine atoms. The **fa** and **cl** cappings are both so small that no steric effects are expected, and both show preference for the structure with missing linkers only. For *E*d,1t the preference is 0.28 eV larger for **cl** than for **fa**, while for *E*d,6t it is 1.07 eV larger for **cl**, which we attribute to the stabilising hydrogen bonding pattern in the 9_h_ missing linker structure (Fig. 2a) which is not formed in the **reo**-structure. Finally, the **e** defect shows a clear preference for the **reo**-structure. This could be a result of strain in the partially dehydrogenated metal clusters of the missing linker structures. It has previously been shown that the metal cluster condenses and becomes asymmetric when the cappings are removed from the **reo**-structure in UiO-66(Hf) by dehydroxylation (to form **e**-type defects).^19^ The partially dehydroxylated metal clusters in our missing linker structures are geometrically distorted from both the hydroxylated and the completely dehydroxylated metal cluster (*c.f.* ESI for details†).

**Fig. 3:**
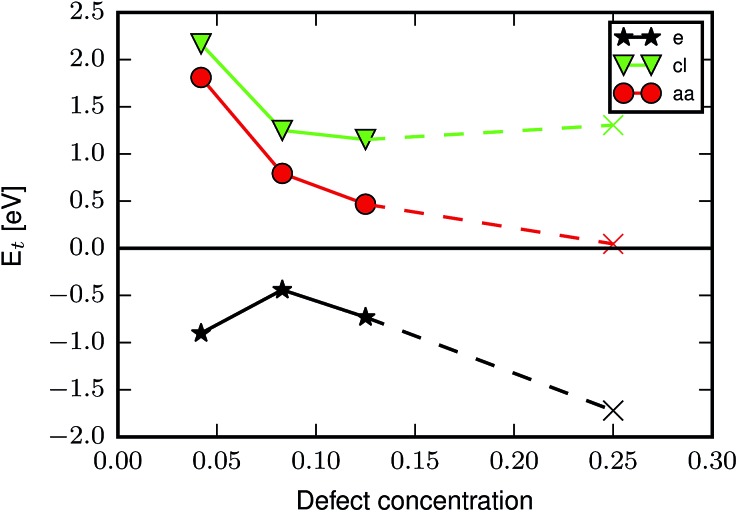
Transformation energy as a function of defect concentration for the **aa** (red circles), **cl** (green triangles) and **e** (black stars) cappings. Circles/triangles/stars mark the concentrations where the best defect configuration has previously been identified by calculating all possible structures,^26^,^39^ while × indicates that only selected configurations of missing linkers were optimised at that defect concentration.

From the results in Table 1 it appears that formation of the **reo**-structure becomes more favourable with increasing defect concentration, since *E*d,1t is generally higher than *E*d,6t, however, since we have not calculated all possible structures with 6 missing linkers the values of *E*d,6t in Table 1 could in principle be higher, if a more stable structure with 6 missing linkers existed. To investigate the concentration-dependence further we calculated the transformation energy for **aa**, **cl** and **e**, using the lowest energy defect structures with two and three missing linkers determined in our previous force field calculations^39^ (**aa**, **cl**) and in ref. 26 (**e**) (see ESI† for schematics of these defect arrangements). The results are plotted in Fig. 3, and show that for **aa** the preference for missing linkers decreases with increasing defect concentration. A similar trend is observed for **cl**, however here the transformation energy plateaus for higher defect concentrations, perhaps because more hydrogen bonds can be formed. The curve for the **e** defect has a different shape, the origin of which we can only speculate about, but formation of the **reo**-structure is always favoured.

The formation of defects has previously been calculated to be energetically unfavourable,^29^,^32^,^39^ however these calculations did not include configurational entropy contributions to the free energy which will lead to an equilibrium population of defects. Furthermore, the concentrations in real samples will be influenced by the kinetics of nucleation and growth. The kinetics can be changed by varying synthesis parameters such as temperature and reaction time, and more intuitively also the ratio of modulator : BDC in the synthesis. The p*K*_a_ value determines the fraction of **tfaa**/**aa**/**fa** modulator molecules that are deprotonated in solution at any given time, and thereby the effective modulator : BDC ratio. In our calculations we consider the different modulators for a given concentration of defects, which would arise from different molar concentrations of modulator during synthesis. The results suggest that in changing the amount of defects (*i.e.* the concentration of the modulator), the ratio between missing linker and missing metal cluster defects is also changed.

### 3.2 Electronic structure

In inorganic semi-conductors a common approach for modulating the optical band gap and electronic properties of a material is to introduce defects.^51^ The question as to if the missing linker phenomenon also modulates the electronic properties of UiO-66 was recently addressed in ref. 26 in the case of missing linkers only, and considering the **e** type defect. It was shown that the introduction of defects leads to a small decrease in the band gap and a decrease in the metal to ligand charge transfer energy, which is favourable for photocatalysis. While the absence of cappings gives easier access to the active sites on the metal cluster, such uncoordinated sites are unlikely to be abundant in solution and we therefore investigate the effect of different cappings on the electronic structure of the material.

**Fig. 4:**
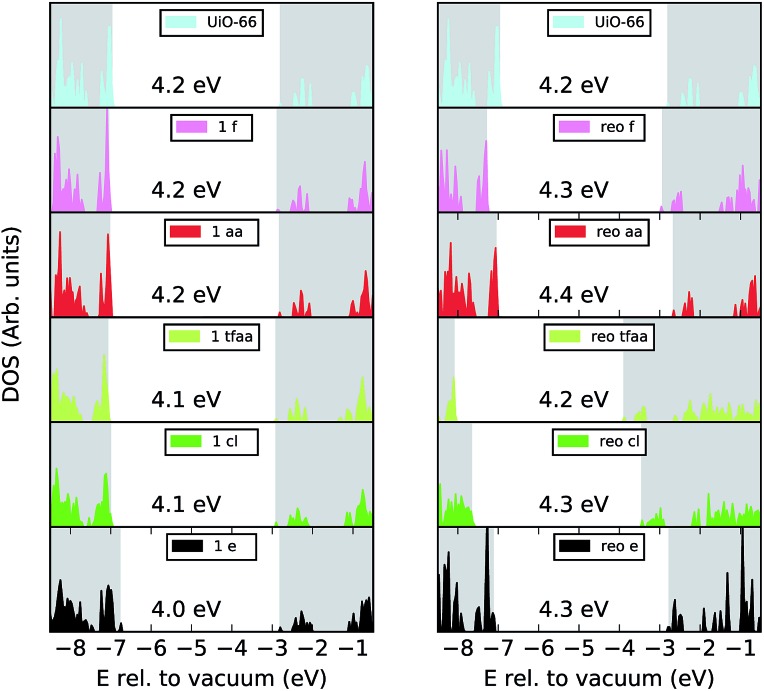
Electronic DOS spectra aligned relative to the vacuum level of perfect UiO-66 (top) and its defect structures with different cappings with 1 missing linker (left) and the **reo**-structure (right). The grey background areas in each plot are the conduction band and valence bands, and the white area between them indicates the band gap.

We calculate the electronic density of states (DOS), band alignment and valence orbital localisation for a single missing linker defect and for the **reo**-structure for the different capping types. The normalised DOS of UiO-66 and its defective structures are shown in Fig. 4. Since the position of the bands relative to the vacuum level is of importance for charge transfer processes (*e.g.* from the framework to a guest molecule or reactant) the plots are aligned relative to vacuum and the position of the band gap is marked by a white background. Our calculations give a band gap of 4.2 eV for defect-free UiO-66 in good agreement with previous calculations at this level of theory (3.9–4.6 eV) and experimental values which range from 3.8–4.1 eV.^12^,^42^,^43^,^52^,^53^ Fig. 4 shows that the band gap variation between defect-free UiO-66 and the defective structures are up to 0.2 eV, with the largest changes observed for the single **e** defect and the **aa**-capped **reo**-structure. It is also clear from the plot that the **aa** and **fa** cappings preserve the shape and energetic position of the DOS, both for the single defect and for the **reo**-structure. This is perhaps not surprising given the similarity between these cappings and the BDC linker. For the **cl**, **tfaa** and **e** cappings some broadening of the highest occupied states are observed, and the **cl** and **tfaa** capped **reo**-structures are down-shifted in energy with respect to the defect-free structure.

**Fig. 5:**
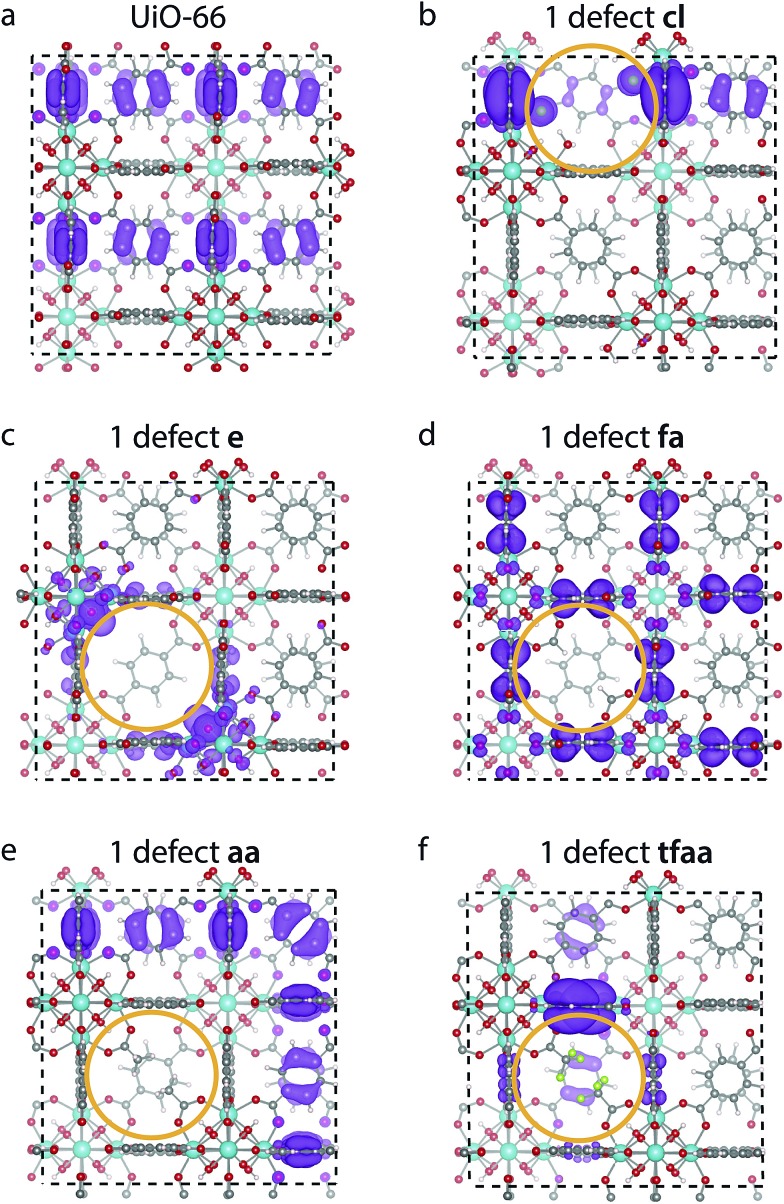
Electron density of the highest occupied band of (a) perfect UiO-66 (top) and UiO-66 with one defect capped by (b) **cl**, (c) **e**, (d) **fa**, (e) **aa** and (f) **tfaa**. The site of the defect is marked by an orange ring, and the isosurface value is 0.00045 e Bohr^–3^ for all images.

The changes in the DOS can be correlated with the changes in the shape of the frontier orbitals. The highest occupied crystalline orbital (HOCO) for perfect UiO-66 and a single missing linker defect with different cappings is shown in Fig. 5. The HOCO of perfect UiO-66 is delocalised over most of the organic linkers (Fig. 5a), but when a **cl** capped defect is introduced it becomes localised, with some weight on the Cl atom (Fig. 5b). The HOCO of the **e** defect likewise becomes localised around the defect, in particular with a high density on the oxygen atoms of the metal cluster (Fig. 5c). In contrast to this the HOCOs of the structures with the **aa** and **fa** cappings are still delocalised over many linkers, but with no weight on the cappings themselves (Fig. 5d and e). Finally, for the **tfaa** capping there is some change in the HOCO, but the density is not located on the defect itself (Fig. 5f). For all cappings the lowest unoccupied molecular orbital (LUCO) is largely unchanged (*c.f.* ESI†).

A similar investigation of the HOCO and LUCO of the **reo**-structure (*c.f.* ESI†) shows that the LUCO is still largely unchanged for all structures. Interestingly, the HOCO of the **cl** structure no longer has electron density on the Cl atoms, but the orbital is not fully delocalised over all linkers. A similar case is found for **tfaa**, while the HOCO of the **aa** and **fa** cappings again look similar to that of the defect-free structure. Only for the evacuated structure is the density still localised around the defect.

## 4 Conclusion

We have compared defect structures containing missing linker defects and missing metal clusters for different charge compensation schemes. Our results show that the preferred type of defect depends on the choice of capping, and is influenced by factors such as steric interactions and hydrogen bonding patterns. The preferred defect configuration is furthermore found to depend on the overall concentration of defects. This implies that the type of defects present in the material can be varied by the identity and concentration of the modulator during synthesis.

For both missing ligand and missing metal type defects with the **cl**, **fa**, **aa** and **tfaa** cappings the electronic properties of the material are largely unchanged, and the main effect of the defects is to increase the porosity of the material. Upon removal of the capping to form an **e** defect, the HOCO becomes partly localised on the defect, indicating that this could be an important site of interaction for guest species. Furthermore, it implies that the excitation from the HOCO to the LUCO changes from a linker-to-linker transition to a metal-to-linker transition, which could affect the lifetime of the excited state.^54^ This type of defect is therefore particularly interesting in terms of changing the electronic properties of the material, however further experimental and theoretical efforts are needed to determine the availability of such open sites under relevant conditions. Finally, we note that first-principles simulations are limited to relatively small cell representations, so that a realistic distribution of defects will require the development of multi-scale simulation approaches for metal–organic frameworks.

## Conflicts of interest

There are no conflicts to declare.

## Supplementary Material

Supplementary information
