# Supplementary material for: Vacancy defect configurations in the metal–organic framework UiO-66: energetics and electronic structure
Source: J Mater Chem A Mater. 2018 Apr 24;6(18):8507–13. doi: 10.1039/c7ta11155j (PMC6003546; doi:10.1039/c7ta11155j)
Supplement: Supplementary file 1 [file TA-006-C7TA11155J-s001.pdf]

Electronic Supplementary Material (ESI) for Journal of Materials Chemistry A.
This journal is © The Royal Society of Chemistry 2018

# Supplementary information for "Defects in the metal-organic framework UiO-66: Energetics and electronic structure"

K. L. Svane,[1, a)] J. K. Bristow,[1] J. D. Gale,[2] and A. Walsh[3]

[1)] *Department of Chemistry, University of Bath, Bath, United Kingdom.*

[2)] *Nanochemistry Research Institute/Curtin Institute for Computation, Department of Chemistry, Curtin University, Perth, Australia*

[3)] *Department of Materials, Imperial College London, London, United Kingdom.*

(Dated: 5 February 2018)

[a)] Present address: Department of Energy Conversion and Storage, Technical University of Denmark, DK-2800 Kgs. Lyngby, Denmark; Electronic mail: kasv@dtu.dk

## A. Defect arrangements for 6 defective linkers

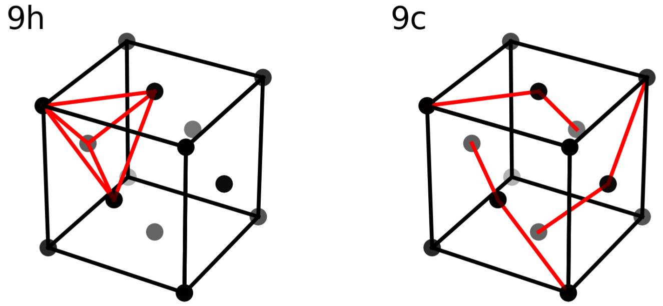


FIG. S1. Schematic representations of the investigated configurations with 6 missing linkers in the cubic unit cell: $(9_h,9_h,9_h,9_h)_{111111111111222}$ (left) and $(9_c,9_c,9_c,9_c)_{3333333333333222}$ (right), following the notation in ref. 1. The black dots represent metal clusters and red lines indicate the positions of missing linker defects.

## B. Defect arangements for 2 and 3 defective linkers

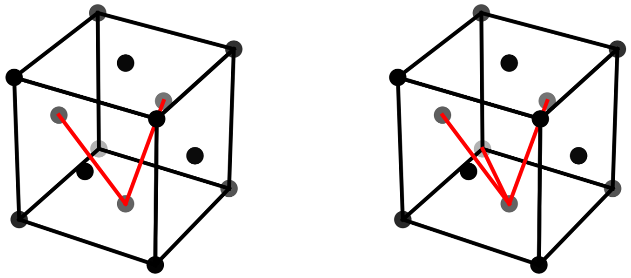

FIG. S2. Schematic representations of the investigated configurations with 2 and 3 missing linkers in the cubic unit cell for the **cl** and **aa** cappings: $(10_d,11,11,12)_1$ (left) and $(9_h,11,11,11)_{111}$ (right), following the notation in ref. 1. The black dots represent metal clusters and red lines indicate the positions of missing linker defects.

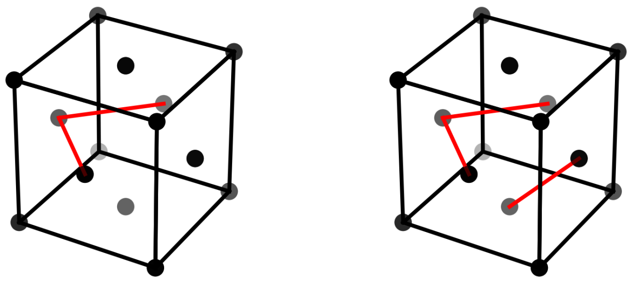

FIG. S3. Schematic representations of the investigated configurations with 2 and 3 missing linkers in the cubic unit cell for the **e** defect: $(10_c,10_c,12,12)_2$ (left) and $(9_b,10_c,11,12)_{233}$ (right), following the notation in ref. 1. The black dots represent metal clusters and red lines indicate the positions of missing linker defects.

## C. Optimised unit cell parameters from the electronic structure calculations

TABLE S1. DFT unit cell parameters of defective UiO-66 structures.

| System | cell parameters (**a**, **b**, **c**) ($\alpha$, $\beta$, $\gamma$) |
|---|---|
| **Experiment**[2] | (20.755, 20.755, 20.755) (90, 90, 90) |
| **non-defective** | (20.74, 20.74, 20.74) (90, 90, 90) |
| **aa capping** | |
| $1_{aligned}$ | (20.79, 20.79, 20.76) (90, 90, 90) |
| $1_{misaligned}$ | (20.79, 20.79, 20.76) (90, 90, 90) |
| 2 | (20.78, 20.81, 20.78) (90, 90, 90) |
| 3 | (20.81, 20.81, 20.81) (90, 90, 90) |
| $6_{9h}$ | (20.84, 20.83, 20.84) (90, 90, 90) |
| $6_{9c}$ | (20.76, 20.76, 20.78) (90, 90, 90) |
| **reo** | (20.74, 20.77, 20.74) (90, 90, 90) |
| **cl capping** | |
| 1 | (20.76, 20.74, 20.78) (90, 90, 90) |
| 2 | (20.76, 20.75, 20.76) (90, 90, 90) |
| 3 | (20.74, 20.74, 20.74) (90, 90, 90) |
| $6_{9h}$ | (20.69, 20.70, 20.69) (90, 90, 90) |
| $6_{9c}$ | (20.77, 20.76, 20.76) (90, 90, 90) |
| **reo** | (20.74, 20.70, 20.75) (90, 90, 90) |
| **tfaa capping** | |
| $1_{misaligned}$ | (20.84, 20.85, 20.70) (90,90,90) |
| $1_{aligned}$ | (20.87, 20.86, 20.68) (90,90,90) |
| $6_{9h}$ | (20.94, 20.94, 20.97) (90, 90, 90) |
| $6_{9c}$ | (20.75, 20.87, 20.75) (91, 90, 91) |
| **reo** | (20.71, 20.72, 20.69) (90, 90, 90) |

TABLE S2. DFT unit cell parameters of defective UiO-66 structures.

| System | cell parameters (**a**, **b**, **c**) ($\alpha$, $\beta$, $\gamma$) |
|---|---|
| **fa capping** | |
| 1 | (20.76, 20.76, 20.77)(90, 90, 90) |
| $6_{9h}$ | (20.74, 20.74, 20.74) (90, 90, 90) |
| $6_{9c}$ | (20.74, 20.74, 20.74) (90, 90, 90) |
| **reo** | (20.74, 20.74, 20.74) (90, 90, 90) |
| **e capping** | |
| 1 | (20.76, 20.76, 20.73) (90, 90, 90) |
| 2 | (20.78, 20.77, 20.66) (90, 90, 90) |
| 3 | (20.79, 20.71, 20.65) (90, 90, 90) |
| $6_{9h}$ | (20.67, 20.67, 20.67) (90, 90, 90) |
| $6_{9c}$ | (20.74, 20.74, 20.74) (90, 90, 90) |
| **reo** | (20.57, 20.57, 20.57) (90, 90, 90) |

## D. Electronic chemical potential variance

TABLE S3. Variance in the average electrostatic potential ($\Phi_{av}(\mathbf{r})$) and UiO-66 and its defective structures when calculating the spherical average with a spherical radius of 1.5 Å.

| | UiO-66 | **cl** | **fa** | **aa** | **tfaa** | **e** |
|---|---|---|---|---|---|---|
| 0 (UiO-66) | 9.93E-7 | - | - | - | - | - |
| 1 defect | - | 2.01E-4 | 5.12E-5 | 1.60E-5 | 2.30E-4 | 1.22E-5 |
| **reo** | - | 1.33E-6 | 1.12E-8 | 3.05E-8 | 9.90E-8 | 1.72E-9 |

## E. Metal cluster geometry for *e* defects

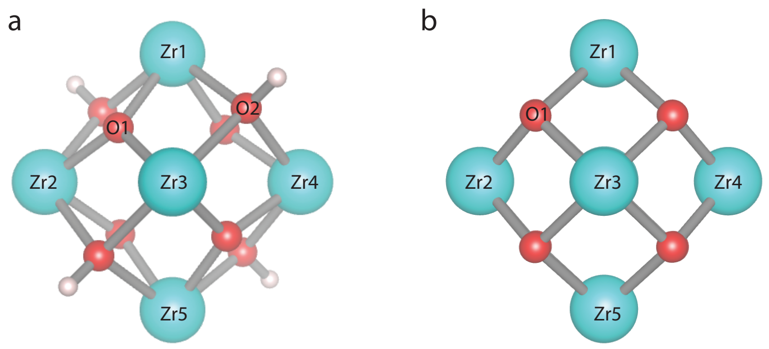


FIG. S4. Structure and labelling of a) the hydroxylated metal cluster and b) the dehydroxylated metal cluster.

TABLE S4. Selected interatomic distances in the metal clusters of perfect UiO-66 and UiO-66 with **e** defects. The atom labels refer to figure S4, and for **e** defect structures the distances are taken from the most defective metal cluster(s) only. For the **reo**-structure two distinct values are given, since this cluster is still highly symmetric, while for the structures with 1-6 defects with many inequivalent distances we give the range of values. All values are in Å.

| | node | Zr1-O1 | Zr1-O2(H) | Zr1-Zr2 | Zr1-Zr5 |
|---|---|---|---|---|---|
| UiO-66 | 12 | 2.07 | 2.26 | 3.53 | 4.99 |
| 1 defect | 11 | 2.01-2.24 | 2.24-2.32 | 3.33-3.57 | 4.91-4.95 |
| 2 defects | $10_c$ | 2.01-2.21 | 2.21-2.29 | 3.29-3.60 | 4.81-4.88 |
| 3 defects | $9_b$ | 2.02-2.18 | 2.19-2.30 | 3.21-3.51 | 4.74-4.79 |
| $6_{9c}$ | $9_c$ | 2.02-2.15 | 2.21 | 3.28-3.48 | 4.76 |
| $6_{9h}$ | $9_h$ | 1.98-2.22 | 2.28 | 3.31-3.60 | 4.79 |
| **reo** | 8 | 2.06/2.15-2.16 | - | 3.29/3.33 | 4.78/4.65 |

## F. Band structures

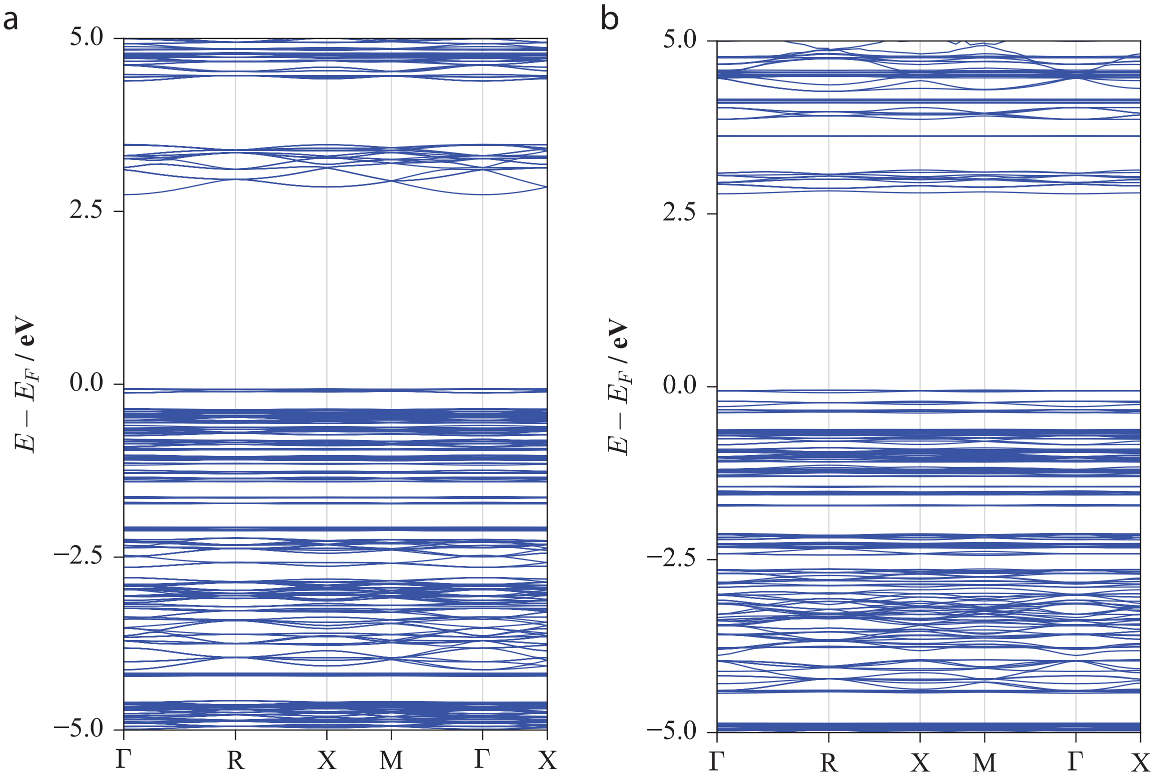


FIG. S5. PBEsol+D3 band structures for a) defect-free UiO-66 and b) the **reo**-structure for the **e** type defect.

## G. Frontier orbitals

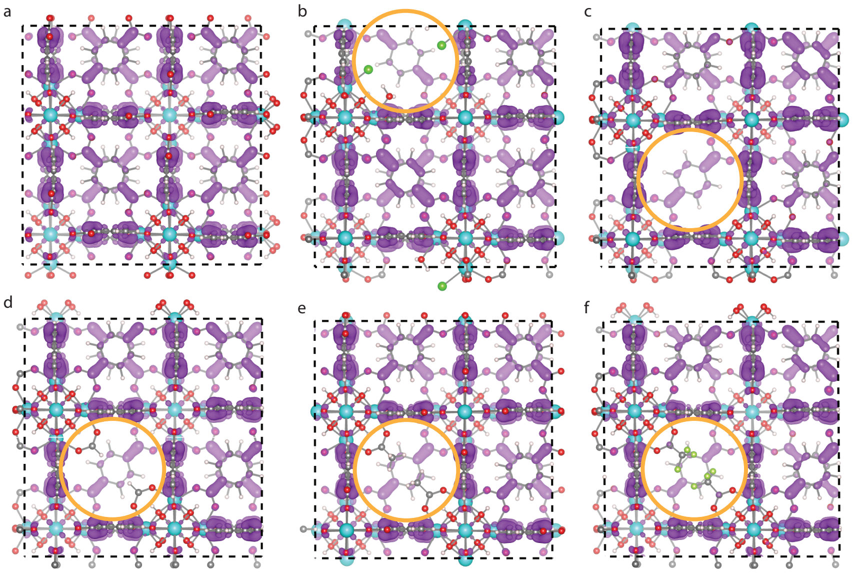


FIG. S6. Lowest unoccupied molecular orbital of a) defect-free UiO-66, and UiO-66 with one defect capped by b) **cl**, c) **e** d) **fa** e) **aa** and f) **tfaa**. The isosurface value is 0.0004e/bohr$^3$ for all structures.

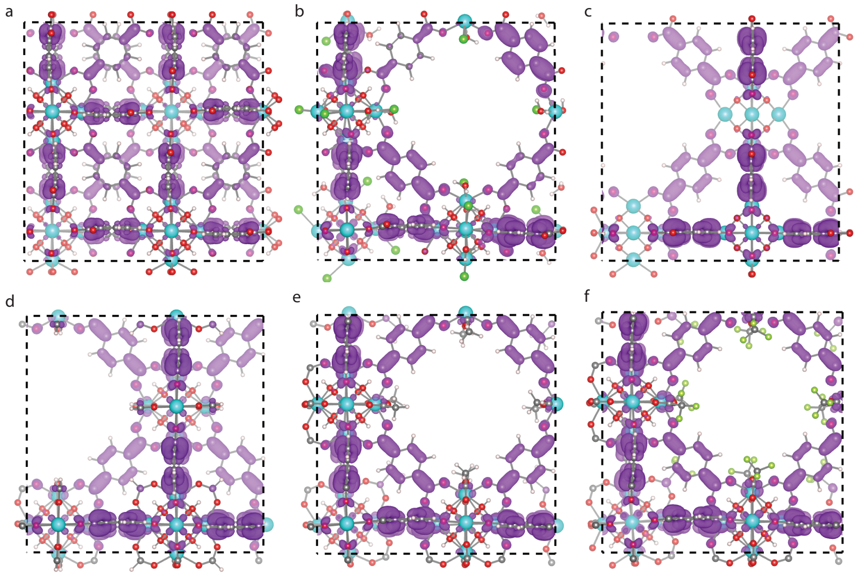


FIG. S7. Lowest unoccupied molecular orbital of a) defect-free UiO-66, and the **reo**-structure capped by b) **cl**, c) **e** d) **fa** e) **aa** and f) **tfaa**. The isosurface value is 0.0004e/bohr$^3$ for all structures.

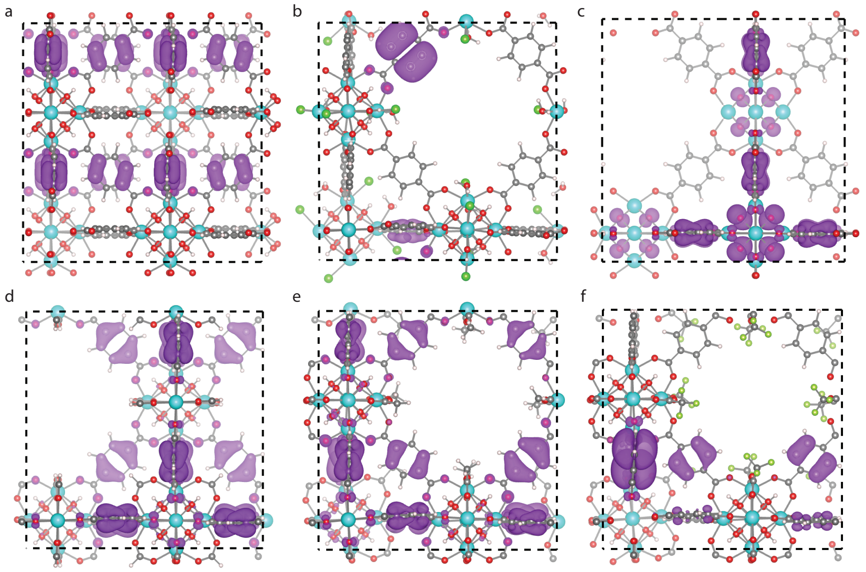


FIG. S8. Highest occupied molecular orbital of a) defect-free UiO-66, and the **reo**-structure capped by b) **cl**, c) **e** d) **fa** e) **aa** and f) **tfaa**. The isosurface value is 0.00045e/bohr$^3$ for all structures.

## REFERENCES


[1] A. De Vos, K. Hendrickx, P. Van Der Voort, V. Van Speybroeck and K. Lejaeghere, *Chemistry of Materials*, 2017, **29**, 3006–3019.

[2] J. H. Cavka, S. Jakobsen, U. Olsbye, N. Guillou, C. Lamberti, S. Bordiga and K. P. Lillerud, *Journal of the American Chemical Society*, 2008, **130**, 13850–13851.
